# Correlation of Metabolic Profiles of Plasma and Cerebrospinal Fluid of High-Grade Glioma Patients

**DOI:** 10.3390/metabo11030133

**Published:** 2021-02-25

**Authors:** Artem D. Rogachev, Nikolay A. Alemasov, Vladimir A. Ivanisenko, Nikita V. Ivanisenko, Evgeniy V. Gaisler, Olga S. Oleshko, Sergey V. Cheresiz, Sergey V. Mishinov, Vyacheslav V. Stupak, Andrey G. Pokrovsky

**Affiliations:** 1 V. Zelman Institute for Medicine and Psychology, Novosibirsk State University, Pirogov str., 2, 630090 Novosibirsk, Russia; evgeniy.gaysler@mail.ru (E.V.G.); oleshko.os@gmail.com (O.S.O.); cheresiz@yandex.ru (S.V.C.); agpok@inbox.ru (A.G.P.); 2 N. N. Vorozhtsov Novosibirsk Institute of Organic Chemistry, acad. Lavrentiev ave., 9, 630090 Novosibirsk, Russia; 3 Institute of Cytology and Genetics of Siberian Branch of Russian Academy of Sciences, acad. Lavrentiev ave., 10, 630090 Novosibirsk, Russia; alemasov@bionet.nsc.ru (N.A.A.); salix@bionet.nsc.ru (V.A.I.); n.ivanisenko@gmail.com (N.V.I.); 4 FSBI “Novosibirsk Research Institute of Traumatology and Orthopedics Named after Ya. L. Tsiviyan”, Frunze str., 17, 630091 Novosibirsk, Russia; smishinov@yandex.ru (S.V.M.); vstupack@niito.ru (V.V.S.)

**Keywords:** glioma, metabolomics, liquid chromatography, tandem mass spectrometry, plasma, cerebrospinal fluid

## Abstract

This work compares the metabolic profiles of plasma and the cerebrospinal fluid (CSF) of the patients with high-grade (III and IV) gliomas and the conditionally healthy controls using the wide-range targeted screening of low molecular metabolites by HPLC-MS/MS. The obtained data were analyzed using robust linear regression with Huber’s M-estimates, and a number of metabolites with correlated content in plasma and CSF was identified. The statistical analysis shows a significant correlation of metabolite content in plasma and CSF samples for the majority of metabolites. Several metabolites were shown to have high correlation in the control samples, but not in the glioma patients. This can be due to the specific metabolic processes in the glioma patients or to the damaged integrity of blood-brain barrier. The results of our study may be useful for the understanding of molecular mechanisms underlying the development of gliomas, as well as for the search of potential biomarkers for the minimally invasive diagnostic procedures of gliomas.

## 1. Introduction

Gliomas, which represent the majority (81%) of malignant brain tumors, are classified using WHO grade criteria into 4 grades based on increasing degree of anaplasia and aggressiveness, with malignant gliomas comprising grades III and IV tumors. The most prevalent glioma types include astrocytoma (WHO grade I–IV), oligodendroglioma (WHO grade II–III), and oligoastrocytomas (WHO grade II–III). High-grade gliomas include glioblastoma (WHO Grade IV astrocytoma), anaplastic (WHO Grade III) astrocytoma, anaplastic (WHO Grade III) oligodendroglioma and mixed (WHO Grade III) oligoastrocytomas [1,2].

The most common type of glioma is glioblastoma, which accounts for 82% of malignant gliomas and has the worst survival prognosis, with only 5% of patients surviving 5 years after diagnosis [1,2]. The 5-year survival rates for Grade III malignant gliomas are significantly higher (~25% for anaplastic astrocytoma, 45% for anaplastic oligodendroglioma and ~50% for mixed oligoastrocytic tumors [1]. A multimodal treatment including surgical removal of tumor with a concomitant/adjuvant radio- and chemotherapy (temozolomide) is the first-line standard-of-care for glioblastoma, the use of which increased the median survival to 15 months vs 12 months with radiotherapy alone [2].

Although the clinical point of view exists that early diagnosis (and treatment) do not improve the outcomes in glioblastoma, an alternative opinion suggests that an early diagnosis and accurate tumor classification must become a cornerstone of an efficient personalized therapy [3]. In fact, the current diagnostic procedure for glioblastoma always results in a late diagnosis, since it relies on neurological tests and neuroimaging. Meanwhile, numerous studies underscore the importance of a maximum surgical removal of tumor mass up to the borders of the healthy surrounding tissue for a better survival and life expectancy of glioblastoma patients [3], which, in case of a brain tumor, is, apparently, less feasible with larger and more advanced late-diagnosed neoplasms.

An early diagnosis of a brain tumor is, thus, an important task for the improvement of treatment efficiency and survival of the patients. The development of minimally invasive approaches for the early detection and classification of tumors is indispensable for choosing a more efficient therapeutic strategy.

The malignant cells including those of brain tumors have been long known to possess metabolic alterations [4,5,6]. These features are due to their enhanced survival and reproduction under the conditions when nutrients and oxygen are scarce, which may be caused by the gene mutations resulting in an altered functioning of different enzymatic pathways [7,8,9]. An apparent difference in the metabolic profiles of normal and malignant cells laid the groundwork for the development of diagnostic methods relying upon the identification of specific disease biomarkers. The study of brain tumor metabolome and the search for biomarkers indicative of brain tumor development and helpful in tumor grading or making the diagnosis of tumor recurrence pose an ongoing challenge, different instrumental approaches to which are being developed.

The cerebrospinal fluid (CSF), which is secreted by the choroids plexus and the meninges, and circulates between brain ventricles and spinal subarachnoid space, is involved in a number of protection, metabolic and drainage functions. Consisting of ~80% of blood-derived and 20% of brain-derived components and being permanently renewed, it maintains the electrolytic and acid-base balance of the brain and supplies the nutrients and signal molecules to the neuronal and glial cells. Meanwhile, it serves as a lymphatic system for the CNS by draining off the wastes and neurotoxic products of brain metabolism. It also provides mechanical support and protection against brain traumas and is involved in intracranial pressure maintenance. Filtration of blood-derived immune molecules, such as the immunoglobulins and cytokines through the blood-brain barrier (BBB) into the CSF ensures the immunological function of the CSF. Finally, the CSF is directly involved in the circadian cycle control via its prostaglandin D2 (PGD2) and prostaglandin-D-synthase (PGDS) system [10].

The CSF is an important diagnostic tool for the search of biomarkers of brain diseases and dysfunctions. A number of published studies is dedicated to the identification of biomarkers of Parkinson’s and Alzheimer’s diseases, multiple sclerosis, etc. [11,12,13,14]. An apparent disadvantage of metabolomic approaches to the CSF analysis is the relative invasiveness of the CSF withdrawal procedure, which requires a lumbar puncture.

A number of published studies presents the metabolomic screening for glioma biomarkers in individual CSF [15,16] or plasma [17,18] samples. Meanwhile, a limited number of studies analyses the relative metabolite content in matched CSF and plasma samples in different pathologies, e.g., Alzheimer disease [19], and neither publication compares the matched CSF and plasma metabolite profiles in the patients with gliomas. Here, we present a wide-range metabolomic analysis of the CSF and blood plasma samples in the groups of glioma patients and conditionally healthy controls, as well as a correlation study of the metabolomic profiles of matched CSF/plasma samples in the individual study subjects.

## 2. Results and Discussion

The plasma and CSF samples were obtained from each participant and analyzed in three replicates. As much as 289 metabolites (Table S1) were screened by HPLC-MS/MS using the approach described by Yuan et al. [20]. This approach consists of a targeted analysis of metabolites in MRM mode using positive/negative polarity switching followed by a relative quantitation of the compounds. After analysis of samples, the resulting chromatograms were integrated and the results (Table S2) were processed using statistical methods. Thus, three vectors constructed using the metabolite peaks and characterizing the metabolic profiles were obtained for each study subject (variation of metabolite peak areas between these replicas is shown in Figure S1).

**Figure 1:**
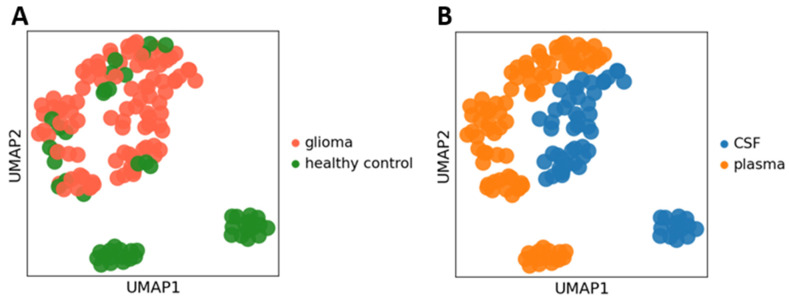
The projection of metabolic profiles onto a 2D space using the Uniform Manifold Approximation and Projection (UMAP). Each point corresponds to a single metabolic profile measurement. (**A**) Points are colored in red and green for glioma patients and control group, correspondingly. (**B**) Points are colored in blue and orange for CSF and plasma samples, correspondingly. PCA and UMAP coordinates were calculated for the log-transformed measurement values using SCANPY python package [22].

The projection of metabolic profiles onto a 2-D space using the Uniform Manifold Approximation and Projection (UMAP) [21] was performed for the primary data analysis and visualization, the results of which are shown in Figure 1. As seen in the figure, the glioma and healthy control metabolic profiles are markedly different for the majority of samples indicating the high diagnostics potential of the method (Figure 1A). Also, a partial separation of profiles was observed between the CSF and blood plasma samples of glioma patients (1B). However, significantly reduced separation of CSF and blood plasma samples of glioma patients may indicate damaged blood-brain barrier. Better separation between each of these pairs of groups was reached using OPLS-DA (see Figures S2 and S3 in Supplementary Materials). To further investigate these observations, we conducted detailed analysis of metabolic profiles.

**Figure 2:**
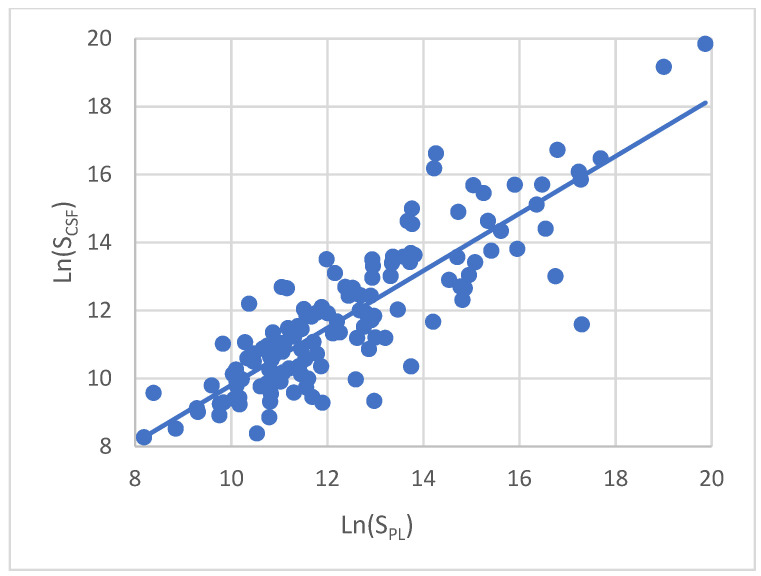
Correlation (R = 0.85) between CSF and plasma metabolite profiles in a combined group of glioma patients and healthy controls. Each point corresponds to an individual metabolite. Ln values of the average peak area of a metabolite in plasma (S_PL_, axis X) and CSF (S_CSF_, axis Y) are shown, respectively.

**Figure 3:**
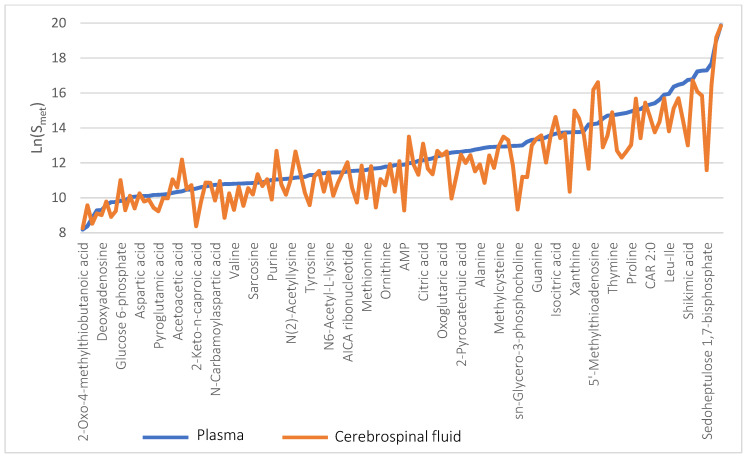
Metabolic profiles of blood plasma (blue line) and CSF (orange line) of the combined group of glioma patients and control group. Metabolites are ranged according to their increasing plasma content. Ln values of the average peak area of a metabolite are shown on the Y axis.

The metabolic profiles of each sample were further averaged across the three replicate measurements. A comparison of mean CSF and plasma metabolic profiles in the combined group of glioma patients and healthy controls shows their high degree of similarity with the correlation coefficient R = 0.85 (Figure 2). The same correlation analysis was performed for the patient and the control groups individually, which did not significantly change the obtained values (R = 0.86 and 0.82, respectively (figures not shown). The profiles of metabolites content in plasma and CSF samples ranked according to their increased representation value in plasma are shown in Figure 3.

Thus, herewith we show that the mean plasma and CSF metabolite profiles are highly correlated to each other across the combined group of glioma patients and healthy controls. However, this analysis does not provide information on the correlation between the content of individual metabolites in plasma and CSF samples.

**Figure 4:**
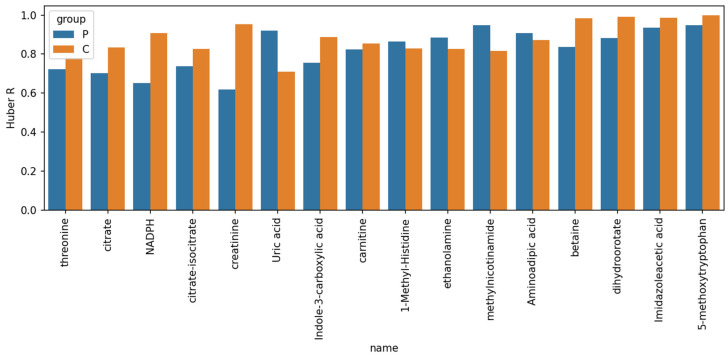
Coefficients of correlation of plasma and CSF metabolite profiles in the two separate groups: the group of patients (P) and control group (C).

In order to find the correlation for each of the analyzed metabolites, we investigated the correlations of their concentrations in plasma and CSF using the regression analysis and Huber’s correlation method [23]. A comparison between the matched CSF and plasma samples of the individual subjects was performed. The correlations were obtained for the combined group of glioma patients and healthy controls, as well as for the groups of patients and controls, separately (see Supplementary Materials for further details). Significant correlations (*p* < 0.05) were observed for 75, 68 and 23 out of 101 studied metabolites in the combined group of glioma patients and healthy controls, or in the patients or the controls groups, separately (see Supplementary Materials). Apparently, the detected sets of metabolites are markedly different with only 16 metabolites being shared by all three groups (see Figure 4).

An analysis of overrepresentation of metabolic pathways from Reactome database performed with a set of 16 found metabolites used as a query (Figure 4) identified 20 statistically significant pathways (FDR < 0.05, see Table S3, isect_pathways). Six out of 20 overrepresented pathways were involved in the transport function of ions, small molecules and other compounds across the cytoplasmic membrane (see Table 1). Additionally, several other metabolic cascades have been identified among the 20 found pathways, as follows: “Neurotransmitter release cycle” (R-HSA-112310), “Citric acid cycle (TCA cycle)” (R-HSA-71403), “Pyruvate metabolism and Citric Acid (TCA) cycle” (R-HSA-71406) и “The citric acid (TCA) cycle and respiratory electron transport” (R-HSA-1428517), which may represent a particular interest for the disease under study.

Also of interest are the metabolites showing highest differences of correlation coefficients between the glioma patients’ group and the controls. The lists of metabolites showing the highest correlation between CSF and plasma samples in the control group, but not the patients group, and vice versa, are shown in Table 2 and Table 3, respectively.

Overrepresentation analysis for the metabolites from the first set (with correlation lost in patients) revealed that the most significant overrepresented pathways with more than one metabolite found were: Metabolism of nucleotides (R-HSA-15869) and Metabolism (R-HSA-1430728).

Almost 70 pathways were found to be overrepresented with 53 metabolites showing plasma-CSF correlation, which is lost in control compared to patients (see Table S3, corr_lostin_control_pathways). These metabolites are frequently found in pathways associated with different kinds of transport, in particular, the transport across the cell membrane.

**Figure 5:**
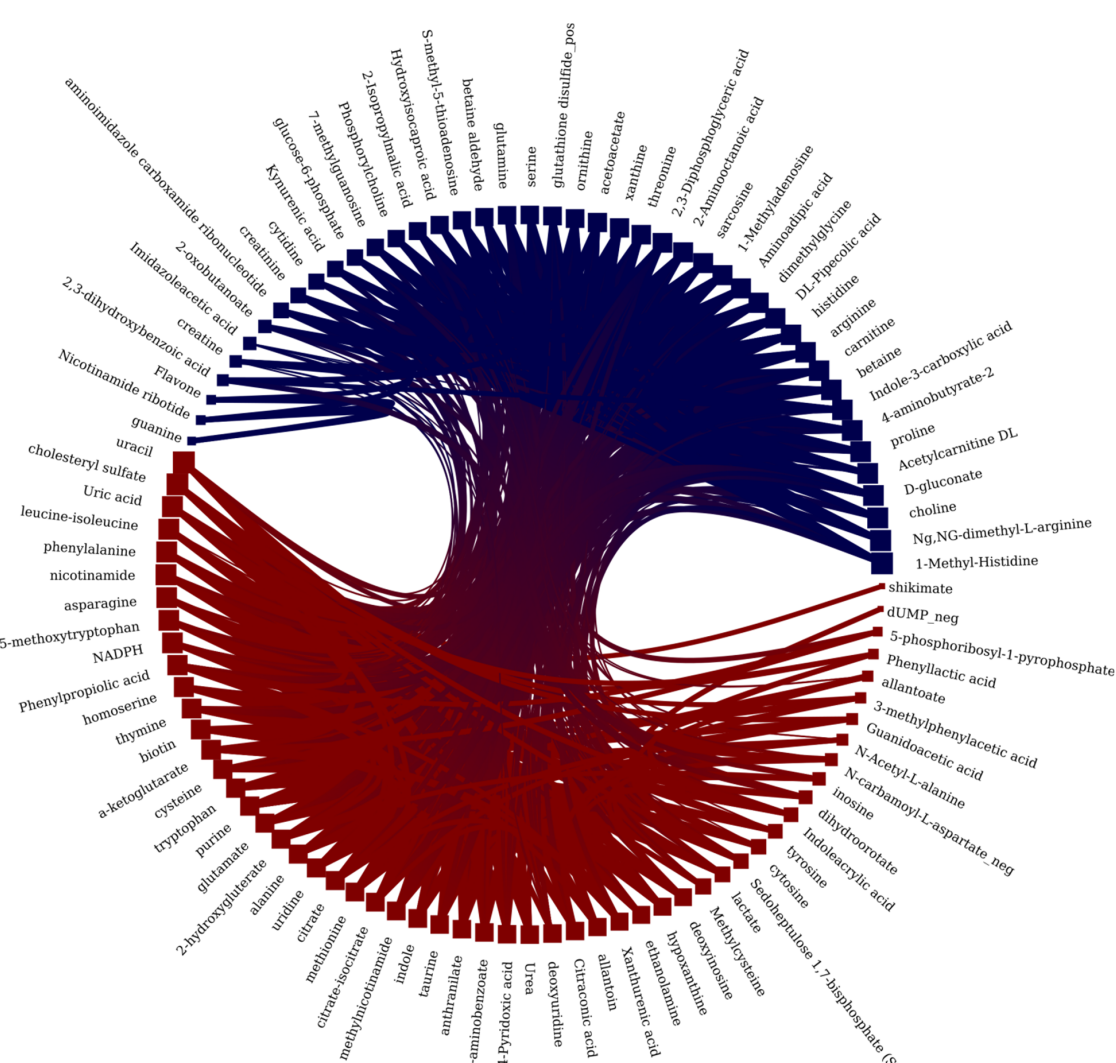
Graph of pairwise robust correlations between the metabolites in CSF or plasma samples. Vertices correspond to metabolites and edges to correlations. Red and blue vertices’ color corresponds to the first and second block inferred from the main graph.

In addition to above analysis, the pairwise correlations between metabolites found in plasma and CSF were studied using the degree-corrected nested stochastic block model [24] (see Figure 5). The graph with 101 vertices and 1115 edges was constructed where each weighted edge corresponds to the correlation with the given correlation coefficient. Two blocks of vertices are distinguished in the main graph of pairwise correlations (corresponding block state entropy was equal to 1856.54). Interestingly, the metabolites from the first block are significantly overrepresented mostly in those Reactome pathways that are associated with transport function (8 pathways among the first 20 ones). In contrast, the metabolites from the second block are significantly overrepresented mostly in the Reactome pathways associated with metabolism, catabolism, and salvage (14 pathways out of 32 found). Detailed tables can be found in Table S3, block_0_pways, block_1_pways.

**Figure 6:**
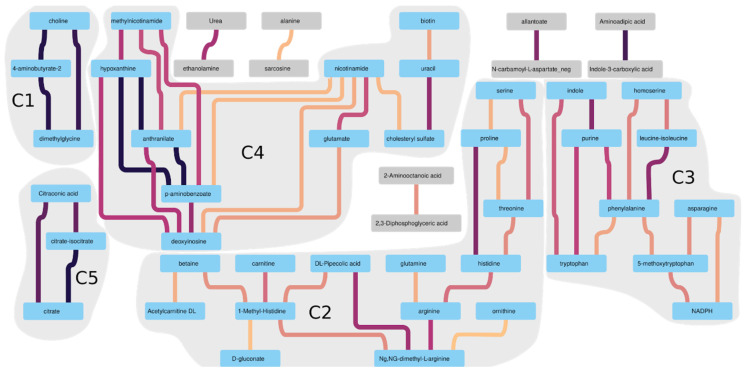
Fragment of the main graph of metabolites pairwise correlation. Only those correlations with correlation coefficients above 95 percentile (R > 0.86) of all correlation coefficients are left. Edge color corresponds to the correlation coefficient (from yellow to black for R ranging from 0.87 to 0.99). Pairs of vertices colored in gray have only a single edge, and vertices colored in blue have more than one edge. The connected components are outlined in gray (entitled C1 to C5).

The metabolites best correlated with each other are depicted by a subgraph of the graph depicted in Figure 5 (see Figure 6 below). This subgraph was obtained after filtering out those edges which have weight below 0.86 (i.e., 95th percentile of all weights). When most of the lower correlating metabolites were filtered out from the subgraph there were five connected components (C1 to C5) with more than a pair of vertices and an edge left. Each of these five connected components has no any edge to other ones. From the interactions found in the subgraph on Figure 6 it is seen that, e.g., citrate, isocitrate, and citraconic acid (all three are from C5) levels significantly correlate with each other in the studied samples. This is probably due to the fact that these three metabolites are involved in the same metabolic pathway(s). To check this hypothesis, an overrepresentation analysis using the Reactome database was conducted for metabolites from each of the five connected components shown in the subgraph above. As expected, metabolites from each connected component had common overrepresented pathways (see Table S3, connected_component_X_pathways, where X is from 1 to 5).

Our analysis supports the idea that the two types of samples under study, namely, plasma and CSF, contain a range of metabolites that change their levels in a correlated manner. The first evidence is the presence of at least 16 metabolites that show significantly correlated levels in plasma and CSF. The second one is that there are pairwise correlations between the levels of a number of metabolites when both samples types are considered together with patients and controls are also pooled. The latter fact suggests the presence of groups of metabolites that might be expected to be involved in common metabolic pathways or are under common regulation. Knowledge of such molecular mechanisms is important for understanding the mechanisms of pathology. There are metabolites from these groups for which there is a correlation between their plasma levels and CSF. Such metabolic groups have been isolated here and can now be further studied.

In our study it was found that the CSF and plasma have a set of metabolites correlated in their content in both matrices. Some of the identified metabolites show an increased correlation in samples from glioma patients compared to the controls, while others show the decreased correlation. We hypothesize that the change in metabolite correlation in patients may be due to differences in blood-brain barrier permeability or active transport of metabolites in glioma patients compared to healthy controls. The increased correlation between CSF and plasma metabolites in patients with gliomas may be due to impaired BBB integrity, leading to an increased correlation between the representation of metabolites in plasma and the CSF. A decreased correlation indicates at a weakening of this dependence, which may be associated with impaired specific transport functions. The molecular mechanisms underlying this distribution of metabolites are of particular interest both for the understanding of the mechanisms of disease development and for building of diagnostic models [25,26].

Currently, there are various hypotheses about the abnormalities of the blood-brain barrier in brain tumors. Tumors are known to compromise the integrity of the BBB, resulting in a vasculature known as the blood-tumor barrier, which is highly permeable and heterogeneous and possessing numerous distinct features including non-uniform permeability and active efflux of molecules [27]. It is also known that tumor cells, including glioblastoma cells, produce extracellular vesicles capable of crossing the BBB [28,29]. At the same time, the content of a number of metabolites in the vesicles produced by GBM cells can differ significantly from their content in cells [30,31].

Numerous literature data show that targeted metabolomic analysis may become an important diagnostic platform in clinical practice in the future. This is facilitated by the comprehensive metabolomic studies based on different platforms (HPLC-MS/MS, GC and GC/MS, NMR, etc.) [32,33], as well as by equipping clinics with modern analytical equipment and developing the bioinformatic methods in systems biology [34,35]. The development of minimally invasive approaches for metabolomics-based diagnostics is an urgent task to be accomplished for the promotion of such techniques, and the choice of plasma instead of CSF as a biological matrix is one of the solutions to this problem.

The application of the described approach made it possible to detect, for example, Alzheimer’s disease at early stages [36,37]. Another study indicates that the metabolomic analysis similar to the one conducted in the current paper can bring forward some ideas to the search of metabolite or peptide markers of Parkinson’s disease by analyzing plasma and CSF samples from 20 patients and 20 healthy controls [38]. As seen from a metabolomic study performed in rats, there is a significant correlation between the levels of steroids extracted from plasma and CSF and their levels in the nervous system [39]. Our analysis not only showed a correlation between a fairly large number of metabolites in plasma and CSF, but also provided preliminary information about the involvement of some metabolic pathways in the development of glioblastoma. Based on these data, we plan to expand the study cohorts and develop analytical methods targeting impaired metabolic pathways.

The use of targeted metabolomic screening by HPLC-MS/MS searches for a large number of metabolites with high sensitivity, so we believe that such studies will provide a more detailed description of the metabolic changes associated with this disease, which may be useful for disease management. The clinical significance of our study lies in the approach to the development of diagnostic methods based on the measurement of metabolites with correlated plasma and CSF content. All of the above suggests the possibility of predicting the diseases of the CNS using plasma metabolome analysis of patients. This will allow future assessment of the metabolomic profile of CSF from plasma sample data, paving the way for the creation of minimally invasive diagnostic methods without CSF sampling.

## 3. Materials and Methods

### 3.1. Study Subjects

Study subjects (Table 4, age distribution of subjects is shown in Figure S6) were enrolled at the Tsiviyan’s Novosibirsk Research Institute of Traumatology and Orthopedics. Only the patients with a confirmed pathomorphological diagnosis of glioblastoma Grade III or IV admitted for surgical resection of tumor were included in the study cohort. The reference group included 11 conditionally healthy donors hospitalized for reconstructive surgery after craniofacial trauma. The diagnosis was confirmed by MRI and the histopathological examination of an excisional biopsy specimen. Glioma patients had not received any drug therapy at the time of their enrollment in the study.

### 3.2. Compliance with Ethical Standards

The study was reviewed and all experimental protocols were approved by the Ethics Committee of the Novosibirsk Research Institute of Traumatology and Orthopedics named after Ya. L. Tsiviyan (No 050/17 of 11.09.2017). The study was registered on ClinicalTrials.gov, Identifier No NCT03865355 (accessed on 21 January 2021) [40]. All procedures involving human participants were found to be compliant with the ethical standards of the institutional research committee and the 1964 Helsinki Declaration and its subsequent amendments or similar ethical standards. An informed consent form was completed and signed by every study subject.

### 3.3. Blood and Cerebrospinal Fluid (CSF) Collection and Processing

The matched blood and CSF samples were collected from fasting subjects on the first day of admission before taking any medications (~7:30–9:30 a.m.). Venous blood was collected into 10 mL BD Vacutainer^®^ KEDTA tubes containing potassium EDTA as an anticoagulant. Plasma was separated from blood cells by 15 min centrifugation at 2000× *g* and 4 °C, aliquoted and kept frozen at −80 °C until further use.

CSF withdrawal was performed by a lumbar puncture at L3–L5 levels using a 19-gauge atraumatic needle. As much as 3 mL of CSF was withdrawn into a 15 mL polypropylene tube, the CSF cells were removed by a 10 min centrifugation, and the CSF samples were aliquoted and kept frozen at −80 °C until further use.

### 3.4. Sample Preparation

All samples were processed at the same time according to the protocol described by Yuan et al. [20]. Briefly, a 100 µL of plasma or CSF sample was precipitated with 400 µL of cooled methanol and incubated overnight at −80 °C for protein precipitation. Then, samples were centrifuged at +4 °C and 16,000× *g* for 15 min. Supernatant was transferred into a new polypropylene tube and dried in a SpeedVac concentrator centrifuge (Thermo Fisher Scientific/Savant, Waltham, MA, USA). Reconstitution was performed in 100 µL of water/methanol (80:20) and subjected to a modified targeted metabolomics analysis with relative quantification. Each sample was analyzed in three replicates.

### 3.5. LC-MS/MS Analysis

Samples were analyzed using a Shimadzu LC-20AD Prominence chromatograph (Shimadzu Corporation, Japan) equipped with SIL-20AC autosampler (Shimadzu Corporation, Japan) thermostated at 10 °C. Sample (10 μL) was injected onto a Prontosil 120-5-Amino column (2.1 × 75 mm) (Econova LLC, Russia). The mobile phase consisted of HPLC buffer A (pH = 9.0, 95% [vol/vol] water, 5% [vol/vol] acetonitrile, 20 mmol/L ammonium hydroxide, 20 mmol/L ammonium formate) and HPLC eluent B (100% acetonitrile), the flow rate during analysis was 0.25 mL/min. The HPLC elution gradient was as follows: from 0 to 3 min, the mobile phase B was decreased from 97% B to 85%; from 3 to 4 min, the percentage of solvent B was decreased from 85% to 30%; from 4 to 10 min, the mobile phase B was decreased to 2% and was kept at 2% for an additional 4.5 min. At minute 14.5, solvent B was increased back to 97% and the column was equilibrated for additional 2.5 min at the flow rate of 0.5 mL/min.

Metabolites (*n* = 289) were analyzed in MRM mode. Data acquisition was performed on API 6500 QTRAP mass spectrometer (AB SCIEX, USA) equipped with an electrospray ionization source operating in the positive/negative switch mode. The main mass spectrometric parameters were as follows. The IS (ion spray) voltages were set at 5500 V and −4500 V for positive and negative modes, respectively. The ion source temperature was set at 475 °C, CAD gas was set as “medium”, Gas1, Gas2 and curtain gas were 35, 35 and 30 psi, respectively. Declustering potential was at ±93V, entrance potential at 10V, and collision cell exit potential at 20 V for positive and negative ion modes. In addition, the polarity switching (settling) time was set to 5 ms, and dwell time was 3 ms for each MRM transition. The precursor ion and fragment ion transitions, the metabolite names, dwell times, and the appropriate collision energies for both positive and negative ion modes were adapted from [20], with several metabolite transitions added by our group. The device was controlled and information collected using Analyst 1.6.2 software (AB SCIEX, Framingham, MA, USA).

### 3.6. Data Processing and Statistics

MRM data were processed in MultiQuant™ 2.1 Software (AB SCIEX, Framingham, MA, USA). Gaussian smooth width was 1.0 point, the minimum peak height was 300 cps, and retention time half window was 30 s. After automated integration, the chromatograms were controlled visually and then the integration results were exported to Microsoft Excel spreadsheet.

The Uniform Manifold Approximation and Projection (UMAP) method was used to reduce the dimension and projection of metabolomic profiles into two-dimensional space [21]. Metabolite set enrichment analysis was carried out using OPLS-DA [41] implemented in pyopls Python library [42].

To assess the relationship between the level of metabolite in plasma and CSF samples, both conventional least squares linear regression and robust regression with Huber’s M-estimates were applied [19]. Comparison was made between plasma and cerebrospinal fluid samples from the same subjects. The correlation coefficient between these two groups of samples, its significance, and residuals were evaluated. The regression residuals were scaled so that the sum of the squares of the residuals for each subject’s samples was equal to one. Additionally, the proportion of total residues attributable to the patient group and the control group was investigated. Correlation coefficient for robust regression was calculated based on weighted least squares model using samples weights obtained from the robust linear models with Huber M-estimates.

Statistical analysis was done using Python and its packages: numpy, scipy, pandas, statsmodels, seaborn, and matplotlib. Graph visualization was done using graph-tool [24] and Cytoscape [43].

## Figures and Tables

**Table 1:** Biological pathways showing overrepresentation with metabolites from a 16-metabolite set with highest correlation between plasma and CSF samples.

Pathway Identifier	Pathway Name	Entities Found	Entities Total	Entities FDR	Submitted Entities Found
R-HSA-425366	Transport of bile salts and organic acids, metal ions and amine compounds	4	165	0.02317	C00719; C00791; C00158; C00366
R-HSA-71291	Metabolism of amino acids and derivatives	4	661	0.02875	C00719; C00791; C00956; C00005
R-HSA-425407	SLC-mediated transmembrane transport	4	418	0.02875	C00719; C00791; C00158; C00366
R-HSA-71403	Citric acid cycle (TCA cycle)	2	50	0.02875	C00005; C00158
R-HSA-549132	Organic cation/anion/zwitterion transport	2	51	0.02875	C00791; C00366
R-HSA-382551	Transport of small molecules	5	967	0.03432	C00719; C00791; C00005; C00158; C00366
R-HSA-71406	Pyruvate metabolism and Citric Acid (TCA) cycle	2	98	0.034312	C00005; C00158
R-HSA-112310	Neurotransmitter release cycle	2	99	0.04432	C00189; C00719
R-HSA-917937	Iron uptake and transport	2	83	0.04500	C00005; C00158
R-HSA-1428517	The citric acid (TCA) cycle and respiratory electron transport	2	233	0.04705	C00005; C00158

**Table 2:** Metabolites with plasma-CSF correlation lost in patients compared to control group. Huber R/p denotes the corresponding R- or p-value of the correlation coefficient for the model with Huber M-estimates.

Name	Patients		Control	
	Huber R	Huber p	Huber R	Huber p
Methylcysteine	−0.12	0.53	0.96	9.75 × 10^−15^
N-Acetyl-L-alanine	0.34	0.10	−0.74	2.82 × 10^−2^
N-carbamoyl-L-aspartate	−0.01	0.98	0.83	1.10 × 10^−2^
deoxyuridine	0.17	0.62	0.97	6.08 × 10^−12^
Acetylcarnitine	0.14	0.72	0.84	2.22 × 10^−3^
4-Pyridoxic acid	0.51	0.07	0.85	1.56 × 10^−3^
S-methyl-5-thioadenosine	0.51	0.09	0.75	2.29 × 10^−2^

**Table 3:** Top 10 metabolites (of 53 total) with plasma-CSF correlation lost in control group compared to patients group. Huber R/p denotes the corresponding R- or p-value of the correlation coefficient for the model with Huber M-estimates. For a full list see Table S3, corr_lostin_control.

Name	Patients		Control Group	
	Huber R	Huber p	Huber R	Huber p
biotin	0.92	7.69 × 10^−31^	−0.27	0.57
phenylalanine	0.86	2.14 × 10^−12^	−0.29	0.54
leucine-isoleucine	0.79	2.05 × 10^−5^	−0.36	0.63
Sedoheptulose 1,7-bisphosphate (SBP)	0.53	4.60 × 10^−5^	−0.52	0.31
hypoxanthine	0.71	9.50 × 10^−9^	−0.24	0.62
cysteine	0.85	1.69 × 10^−7^	−0.04	0.92
creatine	0.50	0.0021	−0.34	0.44
purine	0.84	1.12 × 10^−6^	0.077	0.91
alanine	0.44	0.0071	−0.32	0.49
2,3-Diphosphoglyceric acid	0.85	2.97 × 10^−6^	0.102	0.86

**Table 4:** Age and gender characteristics of control and glioma groups.

Group	Gender (M/F)	Min.	1st Qu.	2nd Qu.	3rd Qu.	Max.	Average	Median	SD
Control	6/5	28.0	52.0	52.0	52.0	77.0	53.0	53.0	12.2
Glioma	11/9	21.0	50.5	50.5	50.5	65.0	55.2	56.0	10.0

## Data Availability

The data presented in this study are available in Supplementary Materials (Table S2).

## Supplementary Materials

The following are available online at https://www.mdpi.com/2218-1989/11/3/133/s1, Figure S1: Relative variation of metabolite levels over three experimental replicas. The notations of the rows (vertical axis) correspond to the individuals (patients and control), those of the columns (horizontal axis) to the metabolites, respectively. Individual code is composed of internal identifier (GBM-X), plasma or CSF (P or L), patient or control (P or C) and positional number, Figure S2: OPLS-DA separation of plasma samples from CSF ones, Figure S3: OPLS-DA separation of glioma patients from control ones, Figure S4: ROC curves for PLS-DA (blue) and OPLS-DA (red) cross-validation of separation between plasma and CSF samples, Figure S5: ROC curves for PLS-DA (blue) and OPLS-DA (red) cross-validation of separation between glioma patients and control, Figure S6: Participants’ age distribution, Table S1: MRM data, Table S2: raw data, Table S3: statistics data.
